# Progress in Medicine

**Published:** 1896-05-30

**Authors:** 


### Progress in Medicine.

### DISEASES OF THE CIRCULATORY SYSTEM.

(*Continued from page* 125.)

**The Nauheim Treatment of Heart Disease.**—While most observers are agreed that the treatment of cardiac disease as conducted at Nauheim is of undoubted value in certain cases, there is a difference of opinion as to the immediate effect produced upon the size of the heart. Both after the baths and the exercises there may be considerable reduction of cardiac dulness. By the most zealous advocates of the system this is thought to indicate a contraction of the diseased and dilated heart down towards the normal. Those, however, who look on the new method of treatment with a somewhat critical eye consider that distension of the lungs and overlapping of the heart by their resonance to be a more probable explanation. Dr. Schott has remarked that if distension of the lungs is the cause of the reduction of dulness, then not only cardiac dulness but liver dulness ought to be diminished from above downwards. Dr. Poore[6] does not think that this by any means necessarily follows. He states that at will he can make his respiration mainly either abdominal or thoracic. When the respiration is thoracic the upper lobes of the lungs undergo extensive dilatation, while the lower lobes remain quiescent. Dr. Poore adds that when a patient is in a bath the pressure of water upon various parts of the body is proportionate to the depth of the part below the surface, and most commonly the abdomen has to bear more pressure than the upper part of the thorax. The inflation of the lungs during respiration will take the path of least resistance and dilation will occur above, while below expansion will be small. Since writing these remarks, Dr. Poore has treated two cases of heart disease with saline baths.[7] Both were cases of aortic valvular disease with enlargement of the heart. He found that after the patients had been in the bath there was considerable reduction of cardiac dulness, but that the apex-beat remained in the same position in one case and moved inwards only about a quarter of an inch in the other. The fixed position of the apex-beat seems to justify the conclusion that diminution of cardiac dulness is due to the advancement of, the anterior border of the left lung over the heart. It is somewhat curious, however, that in one of the cases the circumference of the chest proved to be diminished, instead of increased, as one would expect if the lungs became unusually distended. Dr. Poore offers no explanation of this fact. Dr. Leith,[8] in a valuable paper upon the Nauheim treatment of heart disease, discusses the question of the alteration of cardiac dulness. He mentions that various movements, or other actions such as smoking a cigarette, may materially alter the amount of cardiac dulness in a healthy individual. The varying lines of dulness obtained on percussing the cardiac area of a living subject, and the constant and in great measure correct line obtained on percussing over the heart of the cadaver is worthy of note. The varying dulness noticed in the living does not appear to be altogether attributable to varying inflation of the lungs. A paper by Heitler, of Vienna, is quoted, in which it is stated that the volume of the heart is continually altering while the character of the pulse remains unaffected. Although the dulness may alter, apparently the apex-beat remains in much the same position. Dr. Leith concludes that it appears as if the heart were capable of altering its shape without causing a change in the position of the apex-beat. If such changes of dulness can occur in healthy subjects, due perhaps to alterations in the size of the heart, it follows that, when similar changes of dulness are noticed in patients undergoing some form of treatment for cardiac disease, too much stress should not be laid upon them. While, however, Dr. Leith has drawn attention to these variations of cardiac dulness, he states that his examination of cases treated at Nauheim and others by artificial baths has not led him to conclude that the diminution of cardiac dulness that occurs is due to decrease in the size of the heart. On the contrary, he believes in lung expansion as the cause. Dr. Leith states, however, that it is usual for the apex-beat to be moved inwards; but that this does not necessarily imply decrease in the size of the heart. Dr. Leith has inflated the lungs of the cadaver, and has found that the heart becomes displaced to the right by the distension of the left lung. Interesting though this question of the explanation of the reduction of cardiac dulness is, it is more important to endeavour to find the causes of the improvement that occurs in cases of heart disease. Dr. Leith has gone into this matter carefully. He has tried simple thermal baths, baths containing the various salts of the Nauheim waters, and carbonic acid baths. He considers that the strong saline baths have rather more effect upon the pulse than simple thermal baths, and are more agreeable to the patient. The addition of calcium chloride and barium chloride he does not think has any appreciable effect. The production of carbonic acid gas, however, produces a further diminution in the rate of the pulse and increases its strength. Dr. Leith thinks that the "system" acts mainly by regularly repeated alterations in the blood pressure. The natural ebb and flow between cutaneous and internal vessels is brought more systematically and regularly into play. These changes stimulate tissue metabolism and lead to improvements in general nutrition. The heart participates in these improvements. The alterations in blood pressure are produced partly by the temperature of the baths and partly by the carbonic acid gas. The influence of the salines is probably not great. Dr. Leith thinks that these circulatory changes occur under the influence of the nervous system. The cutaneous nerve endings are stimulated, the cutaneous blood vessels dilate while those in the interior of the body contract. There may be also other effects, such as slight stimulation of cardio-inhibitory and respiratory centres. Dr. Bezly Thorne[9] writes upon self-poisoning in heart disease, and the influence of the Nauheim treatment upon metabolism. He mentions that careful attention must be paid to the intestinal canal in some cases of heart disease, since from that canal toxic products seriously affecting the heart may be absorbed, but he refers also to the beneficial effect upon nutrition produced by the baths and exercises, and mentions as an example of this effect that some patients, who have for some time been unconscious of perspiration, have experienced the return of a normal moisture after undergoing the treatment.

[6] Brit. Med. Journ., Nov. 9, 1895. [7] Ibid., May 9, p. 1,139. [8] Edin. Med. Journ., March, p. 804; Lancet, March 21, p. 757. [9] Lancet, March 21, p. 755.

